# Definition of hourly urine output influences reported incidence and staging of acute kidney injury

**DOI:** 10.1186/s12882-019-1678-2

**Published:** 2020-01-15

**Authors:** Jennifer C. Allen, David S. Gardner, Henry Skinner, Daniel Harvey, Andrew Sharman, Mark A. J. Devonald

**Affiliations:** 1 Nottingham Renal and Transplant Unit, Nottingham University Hospitals NHS Trust, City Campus, Nottingham, NG5 1PB UK; 2 Faculty of Medicine and Health Sciences, School of Veterinary Medicine and Science, University of Nottingham, Sutton Bonington Campus, Leicestershire, LE12 5RD UK; 3 Trent Cardiac Centre, Nottingham University Hospitals NHS Trust, City Campus, Nottingham, NG5 1PB UK; 4 Department of Critical Care, Nottingham University Hospitals NHS Trust, City Campus, Nottingham, NG5 1PB UK

**Keywords:** Acute kidney injury, Urine output, Cardiac surgery, Intensive care, KDIGO

## Abstract

**Background:**

Acute kidney injury (AKI) is commonly defined using the KDIGO system, which includes criteria based on reduced urine output (UO). There is no consensus on whether UO should be measured using consecutive hourly readings or mean output. This makes KDIGO UO definition and staging of AKI vulnerable to inconsistency which has implications both for research and clinical practice. The objective of this study was to investigate whether the way in which UO is defined affects incidence and staging of AKI.

**Methods:**

We conducted a retrospective analysis of two single centre observational studies investigating (i) patients undergoing cardiac surgery and (ii) patients admitted to general intensive care units (ICU). AKI was identified using KDIGO serum creatinine (SCr) criteria and two methods of UO (UO^cons^: UO meeting KDIGO criteria in each consecutive hour; UO^mean^: mean hourly UO meeting KDIGO criteria).

**Results:**

Data from 151 CICU and 150 ICU admissions were analysed. Incidence of AKI using SCr alone was 23.8% in CICU and 32% in ICU. Incidence increased in both groups when UO was considered, with inclusion of UO^mean^ more than doubling reported incidence of AKI (CICU: UO^cons^ 39.7%, UO^mean^ 72.8%; ICU: UO^cons^ 51.3%, UO^mean^ 69.3%). In both groups UO^cons^ led to a larger increase in KDIGO stage 1 but UO^mean^ increased the incidence of KDIGO stage 2.

**Conclusions:**

We demonstrate a serious lack of clarity in the internationally accepted AKI definition leading to significant variability in reporting of AKI incidence.

## Background

Acute kidney injury (AKI) is a rapid deterioration of renal function over hours to days which is associated with adverse clinical outcomes including increased mortality, prolonged length of admission, chronic kidney disease and dialysis dependence [1]. AKI is identified by using rise in serum creatinine (SCr) and/or reduction in urine output as surrogate markers of reduced glomerular filtration rate. Since 2012 AKI has been commonly defined and staged for severity using criteria from Kidney Disease: Improving Global Outcomes (KDIGO) Clinical Practice Guideline for Acute Kidney Injury [2]. The definition proposed by KDIGO includes oliguria, which is defined as urine volume < 0.5 ml/kg/h for 6 h.

Urine output (UO) can detect AKI earlier than SCr, which is recognised to be a late biomarker of AKI e.g. one study suggested that UO can detect AKI 11 h earlier than SCr [3, 4]. In addition it is inexpensive, requiring no laboratory input and can be measured easily by non-specialist staff. UO has been suggested as a sensitive marker of AKI; even very short periods of oliguria can predict subsequent development of AKI (by KDIGO criteria) and SCr rise [5]. Oliguria is also an independent predictor of adverse clinical outcomes [6, 7].. A urine output of < 0.3 ml/kg/h for > 6 h predicts mortality and need for RRT in critically ill patients [5]. The KDIGO cut off of < 0.5 ml/kg/h for > 6 h is liberal in comparison [8]. The use of UO in addition to SCr can improve the ability of KDIGO criteria to predict prolonged hospital stay, RRT or death. A recent study by Howitt et al. demonstrated that patients who met both KDIGO SCr and UO criteria for AKI stage 2 had prolonged hospital stay and increased mid-term mortality versus those who met only the UO criteria [9]. Patients with the same KDIGO stage therefore had different outcomes depending on whether AKI staging was based on SCr, UO or both [10, 11].

The value of using UO to detect AKI may be dependent on the method used to define oliguria, as average UO can differ according to how it is measured and recorded [12]. In most clinical situations, particularly where patients are not catheterised, UO is measured as volume of urine produced over a given period, from which average hourly urine output can be calculated. In critical care environments UO is usually recorded hourly, making it possible to identify each hour where the urine output falls below the KDIGO threshold and whether this persists over consecutive hours. KDIGO acknowledge that there is no consensus on whether UO should be measured using consecutive hourly readings or mean output over a fixed period of time [2]. The method used can affect reported incidence of AKI and sensitivity/specificity of UO as a diagnostic test [12]. It is important to understand the impact that this could have on the reliability of UO for diagnosing AKI. Consistency in definition of UO and oliguria is important. Existing studies have been limited by focusing on single populations and have not considered potential variation across other clinical settings in which AKI is common.

As a retrospective analysis of two single-centre observational studies to investigate novel urinary biomarkers, we investigated patients admitted to cardiac intensive care (CICU) following cardiac surgery or to a general intensive care unit (ICU) to establish if differing methods of measuring UO affected reported incidence of AKI, stratified by stage (Stage 1–3). SCr was used as ‘gold-standard’ for categorising AKI. We calculated sensitivity and specificity for each method to ascertain if either method was preferable in a given clinical setting.

## Methods

We conducted a retrospective analysis of two single-centre observational studies which had been designed primarily to investigate the validity of putative urinary AKI biomarkers. The two study populations were (i) adult patients admitted to CICU following cardiac surgery of any type and (ii) adult patients admitted for any reason to general ICU in a large U.K. teaching hospital. Patients with end stage renal disease were excluded. Ethical approvals were obtained by the Nottingham AKI Research Group as part of a wider programme of research on novel urinary biomarkers for AKI.

Data collection included demographic details, reasons for admission and clinical outcomes including mortality and length of stay. Since all patients were catheterised, urine output (UO) normalised to actual body weight could be measured hourly for up to 48 h (or until death/discharge) and SCr was recorded daily for 5 days. For patients in ICU, UO normalised to ideal body weight was used because, for many of these patients, actual body weight could not be measured. The proportion of patients prescribed diuretics and/or ACEi/ARBs 7 days prior to recruitment was also recorded.

AKI was first diagnosed and staged using KDIGO SCr criteria alone. We then staged AKI according to KDIGO criteria using UO in addition to SCr. KDIGO definition of AKI was an increment in SCr by ≥0.3 mg/dl [≥26.5 mol/l] within 48 h or increase in SCr to ≥1.5 times baseline, which is known or presumed to have occurred within the prior 7 days or urine volume < 0.5 ml/kg/h for 6 h. KDIGO stage 1 was increase in SCr by ≥0.3 mg/dl [≥26.5 mol/l] within 48 h or increase in SCr to 1.5–1.9 times baseline or urine volume < 0.5 ml/kg/h for 6–12 h, stage 2 was increase in SCr to 2.0–2.9 times baseline or urine volume < 0.5 ml/kg/h for ≥12 h, stage 3 was SCr > 3.0 times baseline or initiation of renal replacement therapy (RRT) or urine volume < 0.3 ml/kg/h for ≥24 h or anuria for ≥12 h.

Baseline SCr was established using the methodology of NHS England’s e-alert algorithm [13]. Baseline was determined using pre-existing blood results where available. Where a result was available within 7 days prior to ICU admission/cardiac surgery, the lowest value was taken as baseline. Where a result existed within 365 days but not the preceding 7 days, the median of the results within the past 365 days was taken. Where no preceding result existed a presumed baseline was determined by assuming an eGFR of 75 mL/min/1.73 m2 and back-calculating using the MDRD equation (as endorsed by ADQI) [14, 15].

We compared two definitions of urine output. UO^cons^ used hourly urine output where each consecutive hour met KDIGO criteria. The number of consecutive hours with urine output < 0.5 mg/kg/hr., < 0.3 mg/kg/hr. or anuria was calculated and the highest KDIGO stage reached using these criteria or SCr was applied. UO^mean^ used mean hourly urine output measured for every 6, 12 and 24 h period. The highest KDIGO stage reached using this method or SCr was applied.

We used UO^cons^ and UO^mean^ to diagnose AKI using UO alone as a binary classification test (AKI vs no-AKI) based on KDIGO definition of AKI. We used KDIGO SCr criteria as gold standard for diagnosis of AKI and used 2 × 2 tables of frequencies to calculate biomarker characteristics (sensitivity, specificity, positive predictive value, negative predictive value, likelihood ratio, *P*-value) for each UO method in predicting AKI by SCr criteria. In order to compare levels of agreement between two binomial variables such as an AKI event (yes/no) according to differing criteria (SCr versus UO^cons^ or UO^mean^), levels of positive and negative agreement were calculated according to [16, 17]. Positive agreement estimates the conditional probability that if one of the estimates is positive then the other estimate will also be positive. Negative agreement assumes the converse. If both terms are large, there is arguably less need to compare actual to chance-predicted agreement using a kappa statistic; more information is provided for understanding and improving ratings compared with a single omnibus index. Descriptive data of each patient cohort are presented as mean ± 1SD for continuous variables and number of patients (% of group total) positive for each category. Statistical differences between groups of patients on admission to either cardiac surgery (CS) or intensive care unit (ICU) were assessed by Students t-test (age only) or chi-squared test for categorical data. To assess the statistical significance of the predictive value of serum creatinine or differing methods for calculating urine output as potential markers for AKI, then logistic regression was used (ICU only, as mortality was extremely low in CS for this cohort). Fixed binomial outcomes such as No-AKI vs AKI were fitted with binomial errors, with significance determined after correction for relevant co-variates. These were determined as relevant for inclusion in a multi-variable model if their statistical significance in univariate analysis (i.e. fitted alone) had a *P*-value of ≤0.10. The full final model reports significance of each characteristic with associated Wald statistic and F-probability, after correction for confounders e.g. age, presence of diabetes or not and use of diuretics or not in ICU (Referent categories, 0 were; No-Diabetes or No-AKI or No diuretic use). Statistical significance was accepted at *P* < 0.05. All data were analysed using Genstat v19 (VSNi, Rothampsted, UK).

## Results

### Recruitment

**Fig. 1:**
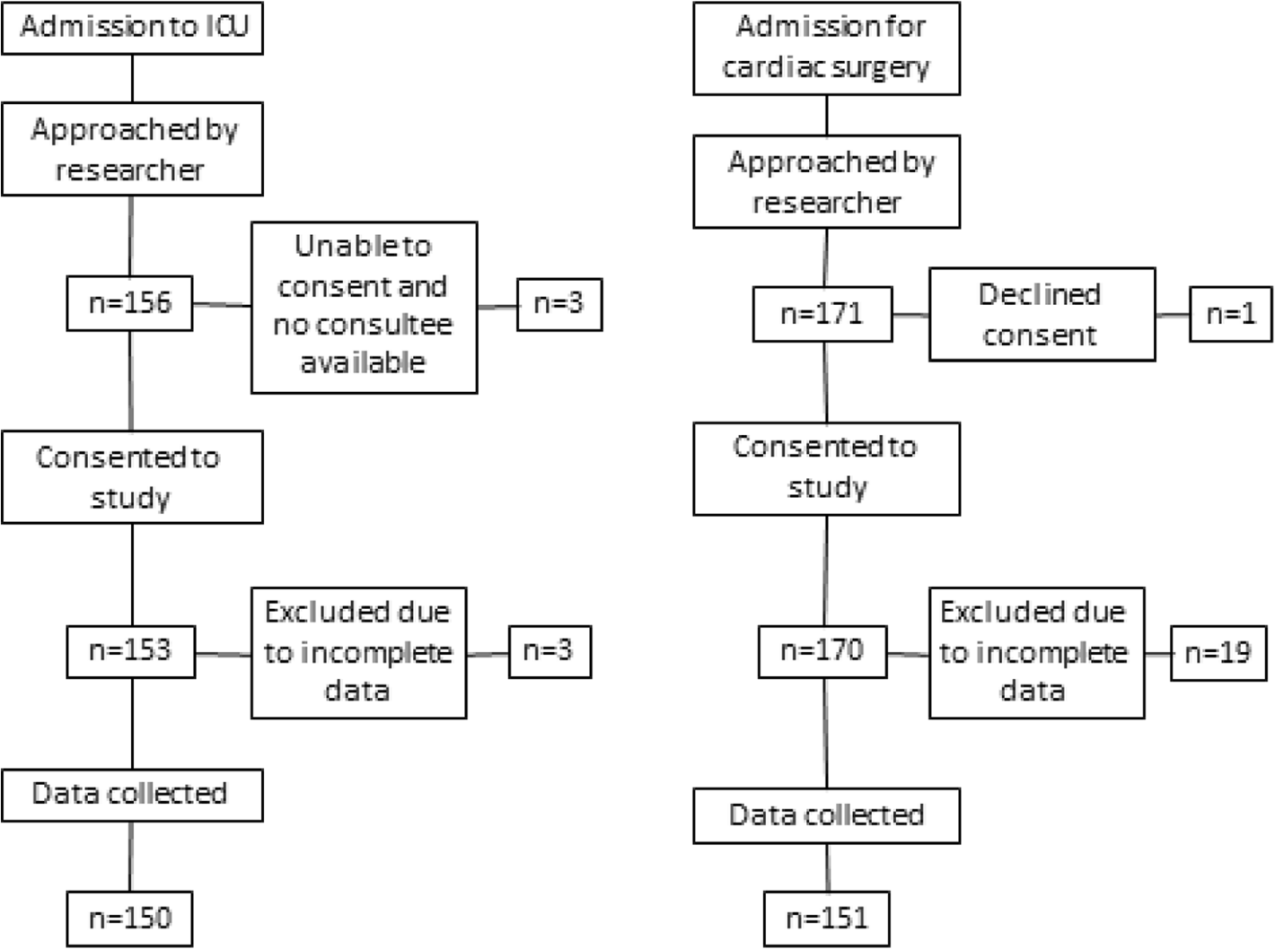
Summary of recruitment to the two studies of novel urinary AKI biomarkers

Recruitment to the two studies is summarised in Fig. 1.

### Patient characteristics

We analysed data from 151 patients undergoing cardiac surgery and 150 patients admitted to ICU (Table 1). Cardiac surgery procedures were valve surgery (45%), coronary artery bypass graft (CABG; 30%), off-pump CABG (11%), combined valve and CABG (11%), aortic root surgery (2%) and other surgeries (1%). 62% were routine procedures and 38% urgent. ICU admissions were medical (34%), neurosurgical (21%), trauma (17%), elective surgical (15%) and emergency surgical (14%). Patients admitted for cardiac surgery in comparison to the ICU group were older (*P* < .001) with a higher incidence of CKD (P < .001) and other co-morbidities (Table 1). Smoking was common in both groups with around half of each group having smoked at some time. Sepsis was significantly more common in ICU (27.3% vs 1.3% in cardiac surgery; *P* < .001)).

**Table 1:** Data are Mean ± 1SD for continuous variables and number of patients (% of group total) positive for each category

Characteristic	Clinical setting		P-value^a^	Mortality as in-patient in ICU only			
	CS *n* = 151 n (%)	ICU *n* = 150 n (%)		Unadjusted OR (95% CI) P-value		Adjusted OR (95% CI) P-value	
Age, yr	68 ± 10	55 ± 17	<.001	1.04 (1.01–1.07)	0.002	1.03 (1.00–1.06)	<.001
Male	110 (73)	96 (64)	0.14	1.38 (0.60–3.17)	0.44	–	–
Smoking (ever)	80 (53)	63 (42)	0.057	0.62 (0.27–1.40)	0.24	–	–
CKD	38 (25)	8 (5)	<.001	1.19 (0.23–6.17)	0.83	–	–
Diabetes	38 (25)	34 (23)	0.03	2.0 (0.87–4.79)	0.10	1.48 (0.58–3.74)	0.22
CCF	36 (24)	5 (3)	<.001	2.45 (0.39–15.3)	0.33	–	–
Hypertension	88 (58)	35 (23)	<.001	1.31 (0.54–3.16)	0.55	–	–
Sepsis	2 (1)	41 (27)	<.001	0.99 (0.41–2.36)	0.99	–	–
Diuretic use	60 (39)	3 (2)	<.001	–	–	–	–
*AKI diagnosis based on*							
SCr alone	36 (24)	48 (32)	0.11	–	–	–	–
Urine output ^cons^	46 (31)	55 (37)	0.25	–	–	–	–
Urine output ^mean^	104 (69)	72 (48)	<.001	–	–	–	–
^**b**^Wald, F-pr for AKI (Model 1)	66.9, 2_*df*_ < .001	15.1, <.001	–	–	–	–	–
^b^Wald, F-pr for AKI (Model 2)	67.3, 2_*df*_ < .001	–	–	–	–	–	–

^a^Statistical differences between groups of patients on admission to either cardiac surgery (CS) or intensive care unit (ICU) were assessed by Students t-test (age only) or chi-squared test for categorical data. ^b^Assessment of the proportion of patients diagnosed as having AKI based on differing clinical criteria (SCr, UO^cons^, UO^mean^) were assessed by logistic regression fitting the outcome (AKI, yes/no) with a binomial error distribution. Wald statistic and F-probability are given after correction for any significant confounders (e.g. Model 1: age & diabetes, Model 2: age, diabetes & diuretic use). Model 2 not conducted for ICU patients as so few were on diuretics. Statistical significance was accepted at *P* < 0.05. SCr, serum creatinine; UO, urine output; urine output ^cons^, output determined by volume produced in consecutive hours; urine output ^mean^, output determined by average volume per hour. All data analyses were conducted using Genstat v17 (VSNi, UK). For mortality data in ICU, referent categories were coded as 0 = No-Diabetes or No-AKI

### Incidence of AKI

Incidence of AKI varied significantly according to the definition of AKI used (Table 2). Based on SCr/RRT alone 23.8% cardiac surgery patients developed AKI (all stages). In ICU, 32% patients developed AKI. The addition of UO to SCr for the diagnosis of AKI significantly increased incidence in both groups, with the larger effect being for patients having cardiac surgery. AKI incidence in cardiac surgery rose from 23.8% using SCr alone to 39.8% using UO^cons^ and to 72.9% using UO^mean^ (*x*^2^ = 78.8 [2_*df*_], *P* < .001). A similar inflation of incidence of AKI was observed in ICU patients rising from 32 to 51.4% using UO^cons^ and to 69.3% using UO^mean^ (*x*^2^ = 42.8 [2_*df*_], *P* < .001).

**Table 2:** Data are number of patients (% of group total) positive for each category. Incidence of KDIGO AKI stages 1–3 in cardiac surgery and ICU was determined using SCr alone vs two methods of measuring urine output (UO). KDIGO stage 1 was increase in SCr by ≥0.3 mg/dl [≥26.5 mol/l] within 48 h or increase in SCr to 1.5–1.9 times baseline or urine volume < 0.5 ml/kg/h for 6–12 h, stage 2 was increase in SCr to 2.0–2.9 times baseline or urine volume < 0.5 ml/kg/h for ≥12 h, stage 3 was SCr > 3.0 times baseline or initiation of renal replacement therapy or urine volume < 0.3 ml/kg/h for ≥24 h or anuria for ≥12 h. UO^cons^ required urine volume to meet KDIGO criteria for each consecutive hour over any 6, 12 or 24 h period. UO^mean^ was mean urine volume meeting KDIGO criteria over any 6, 12 or 24 h period

	SCr/RRT alone		UO^cons^ + SCr		UO^mean^ + SCr	
	Cardiac Surgery (n = 151)	ICU (n = 150)	Cardiac Surgery (*n* = 151)	ICU (*n* = 150)	Cardiac Surgery (n = 151)	ICU (n = 150)
Stage 1	24 (15.9)	22 (14.6)	46 (30.5)	42 (28)	47 (31.1)	29 (19.3)
Stage 2	3 (1.98)	11 (7.3)	5 (3.3)	19 (12.7)	54 (35.8)	55 (36.7)
Stage 3	9 (6)	15 (10)	9 (6)	16 (10.7)	9 (6)	20 (13.3)
All AKI	36 (23.8)	48 (32)	60 (39.7)	77 (51.3)	110 (72.8)	104 (69.3)

### Staging of AKI

**Fig. 2:**
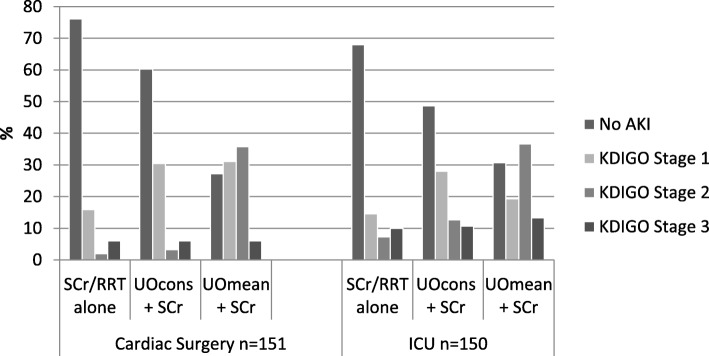
Incidence of KDIGO AKI stages 1–3 in cardiac surgery and ICU was determined using SCr alone vs two methods of measuring urine output. KDIGO stage 1 was increase in SCr by ≥0.3 mg/dl [≥26.5 mol/l] within 48 h or increase in SCr to 1.5–1.9 times baseline or urine volume < 0.5 ml/kg/h for 6–12 h, stage 2 was increase in SCr to 2.0–2.9 times baseline or urine volume < 0.5 ml/kg/h for ≥12 h, stage 3 was SCr > 3.0 times baseline or initiation of renal replacement therapy or urine volume < 0.3 ml/kg/h for ≥24 h or anuria for ≥12 h. UO^cons^ required urine volume to meet KDIGO criteria for each consecutive hour over any 6, 12 or 24 h period. UO^mean^ was mean urine volume meeting KDIGO criteria over any 6, 12 or 24 h period

When UO was used in addition to SCr/RRT to stratify AKI by severity, the proportion of patients allocated to each stage changed considerably for those patients admitted to cardiac surgery compared with those admitted to ICU (Fig. 2). Using SCr alone stage 1 AKI was the most common category in both clinical settings (15.9% in cardiac surgery vs 14.6% in ICU). Incidence of stage 1 AKI doubled in both groups when UO was added to the diagnostic criteria using UO^cons^. In cardiac surgery there was no difference between incidence of stage 1 AKI between UO^cons^ and UO ^mean^ (Fig. 2). In ICU incidence of AKI stage 1 was reduced using UO^mean^ (UO^mean^ 19.3% versus UO^cons^ 28%). Incidence of stage 2 AKI was low in both groups using SCr (1.9% in cardiac surgery, 7.3% in ICU) but rose modestly when UO^cons^ was applied (3.3% in cardiac surgery, 12.7% in ICU). Using UO^mean^ incidence of stage 2 AKI was dramatically inflated, with an increase of 33.8% in cardiac surgery and 29.4% in ICU (Fig. 1). There was no difference in incidence of stage 3 AKI in cardiac surgery when either method of UO measurement was used, with a small rise in stage 3 AKI (2.6%) when UO ^mean^ was used in ICU.

### Sensitivity and specificity of urine output

A comparison between UO^cons^ and UO^mean^ versus SCr/RRT as gold standard for diagnosis of AKI revealed significant differences between the two methods (Table 3). UO^cons^ had reasonable specificity in both groups (79% in cardiac surgery and 73% in ICU respectively) and was therefore good at identifying patients without subsequent SCr rise. UO^mean^ had poor specificity in both groups (36% in cardiac surgery and 45% in ICU respectively) due to a high false positive rate. In cardiac surgery, sensitivity of using UO^mean^ to diagnose AKI was high at 83% with most patients who developed AKI by SCr criteria being correctly identified by UO. In ICU, sensitivity was relatively low at 67%.

**Table 3:** Sensitivity, specificity, positive predictive value and negative predictive value (95% CI) were calculated using 2 × 2 tables of frequencies. KDIGO SCr criteria were applied (Increase in SCr by ≥0.3 mg/dl[≥26.5 mol/l] within 48 h or increase in SCr to ≥1.5 times baseline (which is known or presumed to have occurred within the prior 7 days)) as gold standard for diagnosing AKI. AKI by urine output was defined using KDIGO criteria as urine volume < 0.5 ml/kg/h for 6 h. UO^cons^ required urine volume < 0.5 ml/kg/h each consecutive hour for ≥6 h. UO^mean^ was mean urine volume < 0.5 ml/kg/h over any > 6 h period

	UO^cons^		UO^mean^	
	Cardiac Surgery (n = 151)	ICU (n = 150)	Cardiac Surgery (n = 151)	ICU (*n* = 50)
Number patients with AKI	46 (30.5)	55 (36.7)	104 (68.9)	72 (48)
Sensitivity	0.61 (0.45–0.77)	0.58 (0.44–0.72)	0.83 (0.71–0.95)	0.67 (0.53–0.80)
Specificity	0.79 (0.71–0.86)	0.73 (0.63–0.81)	0.36 (0.26–0.44)	0.45 (0.35–0.54)
PPV	0.48 (0.33–0.62)	0.50 (0.36–0.63)	0.29 (0.20–0.37)	0.36 (0.26–0.46)
NPV	0.87 (0.80–0.93)	0.79 (0.70–0.86)	0.87 (0.77–0.96)	0.74 (0.63–0.85)

### Urine output as a predictor of outcomes

The ability of UO to predict clinical outcomes was assessed by logistic regression in the ICU group alone, due to higher mortality in this group compared with cardiac surgery. In ICU, 11/150 patients died within 72 h, 33/150 patients had died within 30 days and 39/150 had died within 1 year. In cardiac surgery, 0/150 died within 72 h, 5/150 patients had died within 30 days, with no further increase in mortality at 1 year. In univariate models, age was found to be a significant predictor of mortality with presence of diabetes also having a weak confounding effect (*P* = 0.10). Age and diabetes status were thus retained in a multivariate model to assess the predictive ability of UO for mortality (Table 1). For both unadjusted and fully adjusted models, SCr alone was the only significant predictor of mortality for patients admitted to ICU (Table 1).

## Discussion

Using SCr alone, incidence of AKI in cardiac surgery (all stages) of 23.8% was consistent with published studies. A recent meta-analysis covering the period from 2004 to 2014 showed similar incidence of 22.3% (13.6% stage 1, 3.8% stage 2, and 2.7% stage 3) with 2.3% patients requiring RRT [18]. Incidence of AKI in ICU using SCr was lower than published data would predict. The AKI-EPI study looked at multi-national data to estimate incidence of AKI, reporting an incidence of just under 60% in critically ill patients [1]. Incidence of AKI in our ICU population was just 32%. This might be explained by our ICU cohort including 21% neurosurgical patients, as this subgroup is known to have relatively low incidence of AKI compared with general adult ICU patients.

When UO was included in the diagnostic criteria for AKI, incidence rose in both groups. The larger effect was seen in cardiac surgery. There was a significant difference depending on which method of UO measurement was used. UO^cons^ led to a small increase in AKI in both groups. Despite the increase in incidence of AKI using UO^cons^, there was only modest variation from published incidence in cardiac surgery; in ICU incidence rose to a level comparable with published data. When UO^mean^ was applied, AKI incidence in cardiac surgery rose steeply; overall incidence exceeded 70% which is significantly higher than in most published studies. This finding is consistent with results reported by Koeze et al. who found that use of UO together with SCr may increase incidence of AKI by up to 50% [4]. This suggests that UO^mean^ significantly overestimates incidence of AKI in cardiac surgery. A similar inflation of AKI incidence is also present, albeit to a smaller degree, in the ICU group when UO criteria are additionally considered alongside SCr. Taken together, these data suggest that using mean urine output is likely to lead to an over diagnosis of AKI post-cardiac surgery. Although this patient group has been extensively studied with respect to AKI, few studies have included UO criteria for defining and staging AKI. This might explain the absence of this finding in the literature and highlights the importance of using specific and consistent UO criteria.

The impact of using UO was particularly evident when AKI diagnosis was stratified by AKI stage. Both UO^cons^ and UO^mean^ led to an increase in incidence of KDIGO stage 1, but UO^cons^ had little impact on incidence of KDIGO stage 2–3 AKI in either group. Increased incidence of KDIGO stage 1 has less impact clinically because it is associated with fewer and less severe adverse outcomes and is sometimes excluded from large clinical studies of AKI such as TRIBE-AKI [19]. UO^mean^ increased incidence of KDIGO stage 2 AKI in both groups, with the larger effect again being in the cardiac surgery group. This appears to lead to an over diagnosis of KDIGO stage 2 AKI. In ICU this correlated with reduction in the number of people diagnosed with KDIGO stage 1 AKI. This suggests that, as well as leading to over diagnosis of AKI, UO^mean^ may also lead to misclassification as KDIGO stage 2. Furthermore, since urine output is an outcome measure corrected to body weight, then accurate measurement of body weight, rather than an estimation of ‘ideal’ body weight, can also inflate AKI incidence in certain clinical settings such as ICU [20]. Potential consequences of this could include inappropriate initiation of RRT and misclassification in clinical studies of AKI. It is important that this risk is recognised, as mean UO is the only way of measuring UO in the majority of medical patients who do not have a urinary catheter in situ and on wards where UO may be measured less frequently than hourly.

Our results demonstrate that either UO method used independently from serum creatinine was poor at identifying AKI. This is consistent with data from the TRIBE-AKI meta-analysis which found the AUROC for post-operative UO as a marker for AKI was just 0.59 [19]. The use of UO independently from SCr is also inferior at predicting outcomes of length of stay, need for RRT and mortality [9]. Whilst UO^cons^ is less likely than UO^mean^ to over-estimate AKI incidence, the sensitivity is impacted by clinical factors influencing UO such as fluid boluses or diuretics. Patients who are truly oliguric may have a temporary increase in UO which means they no longer meet the consecutive hourly criteria. Absence of oliguria does not itself exclude AKI, as non-oliguric AKI (e.g. contrast induced AKI) is common [12].

The increased sensitivity and high false positive rate of using mean UO may also be influenced by clinical factors such as urinary obstruction or inadequate fluid resuscitation which can affect UO irrespective of renal function or injury. This observation was also made by Ralib et al., who criticised KDIGO UO criteria as being too liberal [8]. In order to reflect glomerular filtration the patient must be adequately hydrated before UO can be useful. The AKIN classification addressed this point but in practice it is difficult to determine “adequate” hydration [21]. Changes in UO can be physiological and not represent disease but rather an auto regulatory response [22]. A study by Solomon in a UK intensive care unit demonstrated that 22% junior doctors had physiological oliguria and were more likely to be oliguric than their patients [23].

The different effects in cardiac surgery and ICU of the two methods of measuring UO suggest that UO is affected by clinical variables in different patient groups. It is important that this is recognised particularly in view of the fact that mean UO is commonly used in most medical settings due to practicalities of patient management (avoiding unnecessary urinary catheterisation), clinical staffing and cost constraints. To our knowledge no previous study has compared the use of UO in ICU with patients undergoing cardiac surgery in order to diagnose AKI.

Limitations of this study included its retrospective design (as part of an observational study investigating novel AKI biomarkers) and the fact that it was conducted in a single centre, although two separate clinical cohorts were studied. Use of SCr as gold standard for AKI definition is a well-documented limitation of most studies of AKI incidence, as SCr is accepted to be a late and poor marker of AKI. In addition, diuretic use was relatively high in the setting of cardiac surgery. Dose and frequency of diuretic administration may confound analyses involving urine output. We have not compared our results with markers of tubular injury or function as ‘biomarkers of AKI’ because these have been validated only in certain clinical settings and are not yet in routine use.

## Conclusions

Our study demonstrates that reported incidence of AKI differs according to the method used to document UO and that the extent of this effect varies between different clinical groups. Clarification of method of UO calculation is important in both clinical and research settings. This single-centre study provides justification for conducting a larger multi-centre study in order to establish more specific criteria for AKI definition.

## Data Availability

Raw data from this study are available from the University of Nottingham ePrints archive at http://eprints.nottingham.ac.uk/

## Abbreviations

AKI: Acute kidney injury
CICU: Cardiac intensive care unit
ICU: Intensive care unit
KDIGO: Kidney disease: improving global outcomes
SCr: Serum creatinine
UO: Urine output

## References

[1] Hoste EA (2015). Epidemiology of acute kidney injury in critically ill patients: the multinational AKI-EPI study. Intensive Care Med.

[2] Khwaja A (2012). KDIGO clinical practice guidelines for acute kidney injury. Nephron Clin Pract.

[3] Dennen P, Parikh CR (2007). Biomarkers of acute kidney injury: can we replace serum creatinine?. Clin Nephrol.

[4] Koeze J (2017). Incidence, timing and outcome of AKI in critically ill patients varies with the definition used and the addition of urine output criteria. BMC Nephrol.

[5] Leedahl DD (2014). Derivation of urine output thresholds that identify a very high risk of AKI in patients with septic shock. Clin J Am Soc Nephrol.

[6] Macedo E (2011). Oliguria is an early predictor of higher mortality in critically ill patients. Kidney Int.

[7] Kellum JA (2015). Classifying AKI by urine output versus serum Creatinine level. J Am Soc Nephrol.

[8] Md Ralib A (2013). The urine output definition of acute kidney injury is too liberal. Crit Care.

[9] Howitt SH (2018). The KDIGO acute kidney injury guidelines for cardiac surgery patients in critical care: a validation study. BMC Nephrol.

[10] Husain-Syed Faeq, Ronco Claudio (2018). The odyssey of risk stratification in acute kidney injury. Nature Reviews Nephrology.

[11] Makris K, Spanou L (2016). Acute kidney injury: diagnostic approaches and controversies. Clin Biochem Rev.

[12] Macedo E (2011). Defining urine output criterion for acute kidney injury in critically ill patients. Nephrol Dial Transplant.

[13] Selby NM (2015). Standardizing the early identification of acute kidney injury: the NHS England National Patient Safety Alert. Nephron.

[14] Bellomo R (2004). Acute renal failure - definition, outcome measures, animal models, fluid therapy and information technology needs: the second international consensus conference of the acute Dialysis quality initiative (ADQI) group. Crit Care.

[15] Gaiao S, Cruz DN (2010). Baseline creatinine to define acute kidney injury: is there any consensus?. Nephrol Dial Transplant.

[16] Cicchetti DV, Feinstein AR (1990). High agreement but low kappa: II. Resolving the paradoxes. J Clin Epidemiol.

[17] Feinstein AR, Cicchetti DV (1990). High agreement but low kappa: I. the problems of two paradoxes. J Clin Epidemiol.

[18] Hu J (2016). Global incidence and outcomes of adult patients with acute kidney injury after cardiac surgery: a systematic review and meta-analysis. J Cardiothorac Vasc Anesth.

[19] Parikh CR (2011). Postoperative biomarkers predict acute kidney injury and poor outcomes after adult cardiac surgery. J Am Soc Nephrol.

[20] Thongprayoon C (2014). Actual versus ideal body weight for acute kidney injury diagnosis and classification in critically ill patients. BMC Nephrol.

[21] Mehta RL (2007). Acute kidney injury network: report of an initiative to improve outcomes in acute kidney injury. Crit Care.

[22] Mehta RL (2013). Acute kidney injury: urine output in AKI--the canary in the coal mine?. Nat Rev Nephrol.

[23] Solomon AW (2010). Urine output on an intensive care unit: case-control study. BMJ.

